# 3D Printed Graphene and Graphene/Polymer Composites for Multifunctional Applications

**DOI:** 10.3390/ma16165681

**Published:** 2023-08-18

**Authors:** Ying Wu, Chao An, Yaru Guo

**Affiliations:** School of Materials Science and Engineering, University of Science and Technology Beijing, 30th Xueyuan Road, Haidian District, Beijing 100083, China; m202120354@xs.ustb.edu.cn (C.A.); m202220400@xs.ustb.edu.cn (Y.G.)

**Keywords:** 3D printing, additive manufacturing, graphene, graphene/polymer composites, multifunctional applications

## Abstract

Three-dimensional (3D) printing, alternatively known as additive manufacturing, is a transformative technology enabling precise, customized, and efficient manufacturing of components with complex structures. It revolutionizes traditional processes, allowing rapid prototyping, cost-effective production, and intricate designs. The 3D printed graphene-based materials combine graphene’s exceptional properties with additive manufacturing’s versatility, offering precise control over intricate structures with enhanced functionalities. To gain comprehensive insights into the development of 3D printed graphene and graphene/polymer composites, this review delves into their intricate fabrication methods, unique structural attributes, and multifaceted applications across various domains. Recent advances in printable materials, apparatus characteristics, and printed structures of typical 3D printing techniques for graphene and graphene/polymer composites are addressed, including extrusion methods (direct ink writing and fused deposition modeling), photopolymerization strategies (stereolithography and digital light processing) and powder-based techniques. Multifunctional applications in energy storage, physical sensor, stretchable conductor, electromagnetic interference shielding and wave absorption, as well as bio-applications are highlighted. Despite significant advancements in 3D printed graphene and its polymer composites, innovative studies are still necessary to fully unlock their inherent capabilities.

## 1. Introduction

In recent years, the exploration of innovative materials and advanced manufacturing techniques has become pivotal in addressing the growing demand for multifunctional materials with tailored properties across a multitude of applications. Among these materials, graphene has garnered exceptional attention due to its remarkable mechanical strength, high thermal and electrical conductivity, and impressive surface area [1]. Graphene-based materials have ignited substantial interest across an expansive spectrum of applications, demonstrating remarkable promise in flexible electronics, energy storage, biomedical devices, and sensors [2]. However, the realization of their full potential often hinges on the ability to engineer intricate structures with precisely controlled properties.

Three-dimensional (3D) printing, or additive manufacturing, is a process of creating 3D objects by adding materials layer by layer based on a digital design or model, which allows for the creation of complex and customized shapes directly from a digital file without the need for traditional subtractive methods such as cutting or drilling [3]. This technique demonstrates great promise in fabricating 3D graphene and graphene/polymer architectures due to its design flexibility, precise placement capabilities, multifunctionality, resource efficiency, and scalability [3]. In addition, printing-induced flow shearing can change the orientations of the high-aspect-ratio graphene sheets, aligning graphene sheets towards the printing direction [4,5]. The alignment of graphene sheets in macroscopic assemblies and composites is beneficial to take full advantage of the exceptional in-plane properties of graphene. These advantages position 3D printing as a promising manufacturing method for harnessing the unique properties of graphene and developing innovative applications in a wide range of industries [6,7,8]. It is worth noting that the field of 3D printing of graphene is still under active research and development. Researchers are continuously exploring new techniques, materials, and applications to harness the full potential of this technology and unlock the possibilities of graphene-enhanced 3D printing.

**Figure 1:**
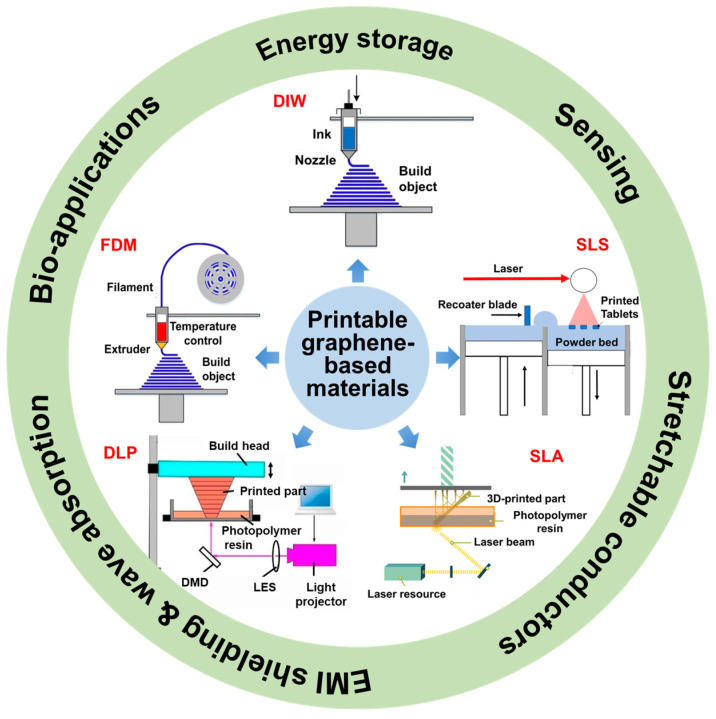
Overview representation of this review [9,10,11,12].

Over the last decade, the field of 3D printing has witnessed a surge in research focused on graphene-based materials, resulting in a diverse array of hierarchical structures with remarkable properties. The extrusion-based, photopolymerization-based, and powder-based 3D printing techniques have been confirmed to be effective in fabricating graphene and graphene/polymer composites [3,9,10,11,12]. Researchers have successfully demonstrated the fabrication of intricate graphene-based architectures with hierarchical features at multiple length scales. These structures include functionalized graphene/polymer composites, porous graphene scaffolds, and graphene-based lattices with controlled porosity. The 3D printed graphene or graphene/polymer composites with rational design of materials and structures have shown their potential in multifunctional applications. In this review, we focus on a comprehensive summary of 3D printing techniques for graphene and graphene/polymer composites, such as printable graphene-based material, the printing apparatus and process, as well as the architecture printed by different 3D printing methods, as shown in Figure 1. We also present recent advances of 3D printed graphene and graphene/polymer composites for several representative multifunctional applications, such as energy storage, sensing, electrical conduction, wave absorption and bio-application.

## 2. 3D Printing Techniques for Graphene and Graphene/Polymer Composites

Typically, the process of 3D printing involves several routines: (i) preparing printable materials; (ii) designing the structures using 3D modeling software, such as computer-aided design software and a 3D scanner; (iii) slicing the 3D model into 2D layers; and (iv) printing materials layer by layer [13,14]. Several 3D printing methods have been developed for additive manufacturing of graphene and graphene/polymer composites, including extrusion techniques (direct ink writing, DIW, and fused deposition modeling, FDM), photopolymerization strategies (stereolithography, SLA, and digital light processing, DLP), and powder-based methods (selective laser sintering, SLS) [7].

The extrusion techniques (DIW and FDM) involve the deposition of material in a controlled manner through a nozzle or extrusion system, building objects layer by layer via extruding and depositing materials in a semi-liquid or molten state. Extrusion techniques offer versatility in terms of material compatibility, as they can accommodate a range of materials such as thermoplastics [15], thermosetting polymers [16], pastes [17], bioinks [18], and even certain metals [19]. While extrusion-based techniques offer several advantages, it is important to note that they may have limitations in terms of architectures complexity and the highest resolution or precision compared to other 3D printing methods [7]. The photopolymerization in 3D printing utilizes light-induced chemical reactions to transform liquid photopolymer resin into solid objects layer by layer. During the printing process, the liquid resin is selectively exposed to ultraviolet (UV) radiation, causing the resin to undergo polymerization and solidification into a desired shape [20]. Photopolymerization-based 3D printing (SLA and DLP) offers several advantages such as high resolution, fast printing speeds, and the ability to produce intricate and precise objects, but limitations in terms of the available material system, printing scale and high equipment cost [21]. Powder-based methods for 3D printing, also known as powder bed fusion, refer to a category of additive manufacturing techniques that utilize powdered materials as the feedstock for fabricating three-dimensional objects. For the fabrication of graphene/polymer composites, it is applicable only to thermal plastic polymer-bound composites [22].

### 2.1. Extrusion Techniques

#### 2.1.1. Direct Ink Writing (DIW)

DIW enables the precise deposition of functional inks to create complex and customizable 3D structures. In DIW, a viscous ink is dispensed through a nozzle or similar extrusion system and is directly written onto a substrate or previously printed layers [23]. Inks for DIW should have a carefully controlled viscosity that allows for controlled extrusion through the printing nozzle while maintaining shape fidelity during deposition [24]. The viscosity should be tailored to the specific printing setup, including nozzle size, extrusion pressure, and printing speed, to achieve optimal flow behavior and avoid issues such as clogging or excessive spreading of the ink. The rheological characteristics, including viscosity and shear-thinning behavior, of graphene- or graphene oxide (GO)-based inks enables them to be extruded and shaped into desired geometries [25].

**Figure 2:**
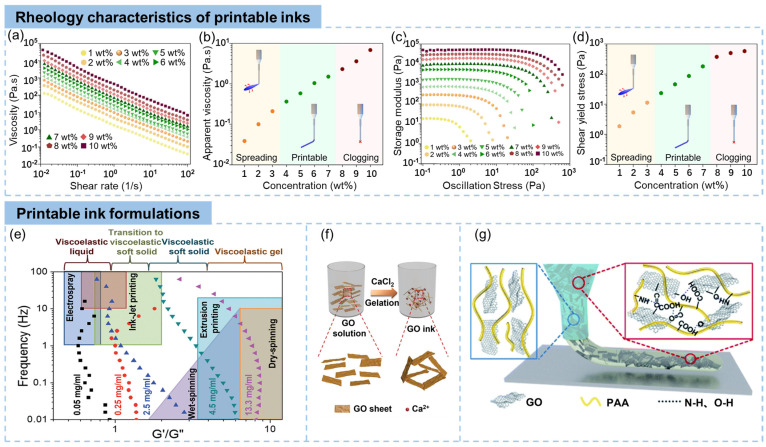
(**a**–**d**) Requirements and characteristics of the rheological properties of printable inks [28], and typical innovative formulations to form 3D printable inks: (**e**) the control of GO concentrations [29], (**f**) the gelation of GO suspensions by adding ions [30], (**g**) the rheological adjustment by adding polymers [31].

GO-based inks are the most used inks for DIW printing of graphene-based materials. The printability of inks is significantly related to their flow properties, including shear thinning behavior and shape retention capacity [26]. The GO aqueous suspension is typically a non-Newtonian fluid with shear-thinning behaviors with a wide range of storage modulus (G′) and loss modulus (G″) that are dependent on the concentration and lateral size of GO sheets [27], making it applicable for DIW-based 3D printing. By controlling the concentration and the lateral size of GO sheets, the rheological properties of GO inks can be effectively adjusted to meet the requirements of DIW. Tran et al. reported a typical work on controlling the viscosity of graphene-based inks by controlling the concentration [28]. As is shown in Figure 2a, the ink exhibited a typical shear-thinning phenomenon (decreased viscosities at higher shear rates), and an enhanced viscosity with increasing concentrations. The apparent viscosity at a shear rate of 100 s^−1^ (Figure 2b) clearly illustrated the dependence of the printability on the viscosity of inks, which suggested a proper viscosity region for 3D printing.

The shape retention capacity of 3D printed structures is predominantly related to the storage modulus and the yield stress at the deposited state [32]. The minimum storage required to maintain printed shapes is determined by [33]:

(1) $$G^{\prime }=1.4\omega {\left(\frac{L}{D}\right)}^{4}D$$

where *ω* is the specific weight of inks, and *L* and *D* represent the length and diameter of the overhanging printed filament, respectively. The minimum shear yield strength ($\sigma_{y}^{sh}$) to achieve precise printing is expressed as [34]:

(2) $$\sigma_{y}^{sh}\geq \frac{\gamma }{R}+\rho gh$$

where *γ*, *R*, and *ρgh* are the surface tension of inks, the diameter of nozzle, and the gravity on printed filaments, respectively. According to these theories, Figure 2c,d clearly show that a minimum shear yield strength is necessary for shape maintenance after printing [28]. Therefore, it is critical to study the rheological properties of DIW inks before printing to optimize the printability.

Though GO dispersions are widely applied as inks for DIW, not all of them are suitable. Several techniques have been developed for the optimization of GO inks, including strengthening GO networks by (i) increasing strengths of inks with a higher concentration and larger lateral size, (ii) gelling induced by inter-sheet crosslinking, and (iii) adding rheology modifying additive [32]. By careful control of viscosity and dynamic modulus of GO suspensions, printable inks can be prepared.

The mechanical properties and viscosity of colloidal GO dispersions are related to the colloid volume concentration. Generally, four different states of GO suspensions were observed with different GO concentrations: viscoelastic liquid, transition region, viscoelastic soft solid, and liquid crystalline gel, as shown in Figure 2e [29]. When the G′ dominates (low GO concentration), the GO suspension is suitable for high-rate processing methods, where the dispersion must spread on contact with the substrate (such as spin coating). When the G″ is higher (higher GO concentration), the rheological property of GO suspensions is suitable for fabrications where the dispersion needs to keep its original shape (such as DIW-based 3D printing) [29,35,36]. At a high GO concentration of 13.3 mg/mL, the G′ was significantly larger than the G″ [29], producing elastic gel-like GO suspensions, which made the GO ink printable under shear stress. In addition to the higher concentration, the larger lateral size of GO sheets also optimizes the GO suspensions to meet the requirements of DIW inks. Ma et al. [37] showed higher G′ and yield strength of GO suspensions containing the same concentration of larger-size GO sheets. With a concentration of 2 vol%, the GO inks containing GO sheets with a large average lateral size of 50 μm delivered a G′ and yield strength of approximately 10^4^ and 100 Pa, respectively, which are suitable for DIW-based 3D printing.

The inter-sheet crosslinking significantly increases the viscosity of GO dispersions, making them applicable for DIW. The addition of metallic cations [38] and ammonium ions [39] lead to crosslinking of GO sheets. For example, Ca^2+^ acts as crosslinkers of GO sheets, turning the GO sol into a viscous hydrogel, as shown in Figure 2f [30]. The addition of Ca^2+^ ions lead to changes in the rheological behaviors of the GO solution, increasing its viscosity and storage modulus. This allows the hydrogel to maintain stability and flow, making it suitable for use in DIW for 3D printing [40]. Note that this gelation method can also be extended to other multivalent ions such as Mg^2+^, Zn^2+^, and Fe^3+^ for the fabrication of printable GO inks [41]. The incorporation of gluconic-δ-lactone and urea generates ammonium ions that interact with GO sheets by hydrogen bonds and influence the rheological properties of the suspension [39].

Adding functional additives, such as polymers [42,43,44] and inorganic particles [45], to adjust the rheological properties of GO suspensions is another common strategy to prepare printable graphene inks. Yuan et al. reported a poly (amic acid) (PAA) functionalized graphene ink [31], as shown in Figure 2g. Both the storage modulus (G′) and the loss modulus (G″) increased after PAA addition, increasing the viscosity of GO dispersions. The platform for G′ and G″ achieved 2500 and 5500 Pa, respectively, due to the PAA-induced crosslinking network, fulfilling the requirements of 3D printing. When the cellulose viscosifier, which forms a hydrogel in water through physical crosslinking due to the numerous hydroxyl groups, was added into the GO/MoS_2_ suspension, both the modulus and yield stress were enhanced, achieving sufficient stiffness to hold the shape after deposition [46].

**Figure 3:**
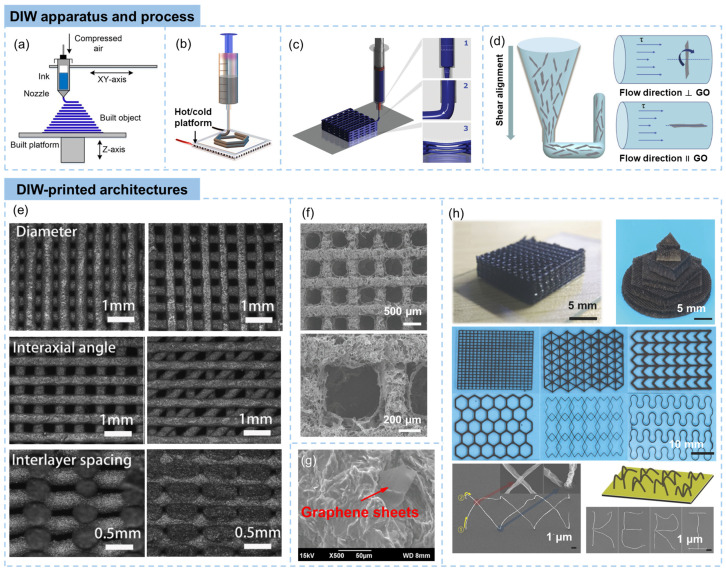
3D printing of graphene using DIW techniques. DIW apparatus and process: (**a**) schematic illustration of the DIW [9], (**b**) DIW on a hot/cold platform [48], (**c**) main steps of the DIW process: (1) ink flow inside the syringe barrel and nozzle, (2) extrusion of the ink from the nozzle and (3) deposition onto the substrate to form a freestanding structure [50], (**d**) shear-induced alignment of graphene sheets during the DIW process [51], (**e**) Patterned structures with different filament diameters, interaxial angles, and interlayer spacing [51], microstructures of filaments in DIW-printed (**f**) graphene aerogel lattices [33], and (**g**) graphene/polymer composites [53]; and (**h**) DIW-printed architectures [33,54].

The apparatus for DIW mainly consists of the extrusion system, ink reservoir, build platform, and soft system. The extrusion process is typically controlled by the pressure of compressed air conducted on the syringe barrel. The nozzle moves back and forth in the x and y axes (and possibly the z axis) with ink extrusions to build the 3D structure layer by layer, as shown in Figure 3a [9]. With careful design of the nozzle or the extrusion system, skeletons with different diameters, core/shell and dual-core structures can be obtained [47]. A hot or cold platform can be integrated into the DIW system (Figure 3b). A cold sink of a temperature far below the freezing point of water functions as a medium to rapidly freezes aqueous GO suspensions [48]. The cold-platform-assisted DIW is applicable to print low-viscous Newtonian GO suspension, as the ejected GO suspensions are rapidly frozen to maintain the structural integrity [49]. With the help of a hot platform or hot environment, solvents evaporate quickly after extruded out from the nozzle, making the vertical writing of GO frameworks possible. The DIW process is generally divided into three steps: (1) ink flow inside the syringe barrel and nozzle, (2) extrusion of the ink from the nozzle and (3) deposition onto the substrate to form a freestanding structure, as shown in Figure 3c [50]. During the extrusion process, shear stresses are imposed to the ink because of the flow. When the graphene sheets with a high aspect ratio are perpendicular to or lying with an angle to the flow direction, the graphene sheets are rotated because of the non-uniform shear stress, as shown in Figure 3d [43,51,52]. This is well known as shear-induced alignment, enhancing mechanical, electrical, and thermal properties along the printing direction.

The sequential deposition of material in discrete layers during the DIW process results in closely packed material units that are aligned along the plane of deposition. By controlling the printing parameter, patterned structures with various filament diameters, interaxial angles and interlayer spacing can be fabricated, as shown in Figure 3e [51]. If without consideration of exceptions, the filaments in DIW-printed structures are generally applied in depositing planes, as the strength of printable inks are usually not high enough to support vertical structures. The freeze-drying technique is usually applied to the direct ink wrote GO objects to prevent the 3D structures from severe shrinking under capillary pressure [55]. Lattices post-processed by freeze drying exhibit porous filaments (Figure 3f). On the other hand, curing is necessary for DIW printed graphene/thermoset polymer composites, producing solid filaments with dispersed graphene sheets, as shown in Figure 3g [53].

Typical architectures printed by DIW techniques are shown in Figure 3h [33,54]. The DIW method enables the creation of 3D freestanding graphene frameworks and intricate graphene/polymer composites. Notably, self-supporting 3D graphene oxide (GO) woodpiles and intricate lattice structures have been successfully engineered using nozzles with internal diameters in hundreds of micrometers [33]. Despite some degree of filament expansion, the GO filaments uphold precise and consistent 3D attributes due to the distinctive rheological characteristics of the inks employed. Though difficult, vertical growth of the printing can be achieved by rapid evaporation of solvents during the DIW process. For example, the attainment of vertical growth during printing at room temperature, which was facilitated by the rapid solvent evaporation during the DIW process, was achieved by Kim and coworkers [54]. It was realized by locally growing GO at the meniscus created at the tip of a micropipette. Freestanding GO structures can be created in a variety of forms, such as bridges, straight wires, suspended junctions, and woven structures depending on the control of the micropipette, as shown in Figure 3h.

#### 2.1.2. Fused Deposition Modeling (FDM)

FDM, also known as fused filament fabrication, is an additive manufacturing technique that uses a filament of thermoplastic material as the feedstock to create three-dimensional objects layer by layer [56,57,58]. Common thermoplastics used in FDM include acrylonitrile butadiene styrene (ABS) [59], polylactic acid (PLA) [60], polyethylene terephthalate glycol [61], and polycarbonate [62]. During FDM of graphene-based architectures, the graphene/polymer composite filament is fed through a heated nozzle which melts the material [63]. The molten material is then extruded onto a build platform or previously printed layers, where it quickly solidifies and forms the desired shape [64,65]. FDM is commonly used for rapid prototyping, functional part production, and educational purposes due to its relative ease of use, low cost, and wide range of available materials [66]. It offers versatility in terms of material selection, colors, and layer resolutions, allowing for the creation of objects with varying mechanical, thermal, and aesthetic properties [67].

**Figure 4:**
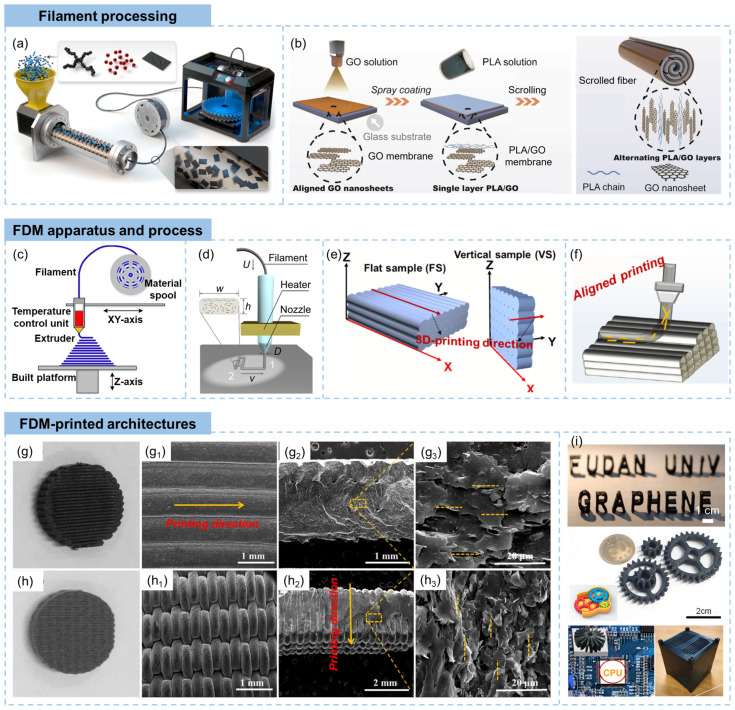
The FDM technique for 3D printing. Filament processing: (**a**) preparation of graphene/polymer filaments via the screw-extrusion method [68], and (**b**) schematic diagram of scrolled fiber with highly aligned GO for FDM [74], FDM apparatus and process: (**c**) schematic illustration of the FDM [9], (**d**) the typical FDM process of graphene/polymer composites which contains (1) an extrusion and (2) a deposition process [75], (**e**,**f**) aligned printing of flat and vertical samples [76], FDM-printed graphene/polymer composite architectures: (**g**,**h**) microstructures [76] and (**i**) macroscopic photos [68,72,76,77].

The graphene/polymer composite filament is generally prepared via a multi-extrusion process, as shown in Figure 4a [68]. In the graphene/polymer filament fabrication process, graphene powders, such as graphene nanoplatelets, graphene oxide, or functionalized graphene, are blended with thermoplastic polymer pellets using a twin-screw extruder [69]. The screw design of the extruder is instrumental in promoting effective mixing and dispersion of graphene within the polymer matrix [70]. The extruder applies high shear forces and temperature, facilitating the dispersion and intercalation of graphene within the polymer. Subsequently, the molten composite material is extruded through a die or orifice located at the end of the extruder [71]. This shaping process gives the material its filament form, with the desired dimensions. To solidify the composite and maintain the filament’s shape, it is rapidly cooled using air- or water-cooling mechanisms, which ensures that the composite solidifies quickly and preserves the desired filament dimensions [72]. In addition to the solid mixing process, graphene/polymer mixtures can also be obtained by dispersing graphene precursors in polymer solutions, followed by solvent evaporation [73]. The mixture is then cut into small pieces and fed to the screw extruder for filament fabrication. A scrolling method for the fabrication of polymer composite filaments with highly aligned GO sheets was reported by Qian et al., as shown in Figure 4b [74]. This process started with the deposition of GO flakes on a preheated glass substrate using a spray coating method, followed by drop casting of PLA solutions to create a well-dispersed composite. The composite film was scrolled into an Archimedean spiral fiber, obtaining 3D printed scrolled fiber filaments.

The apparatus of FDM includes several key elements: (i) a temperature control unit that heats the filament to a temperature at which it melts and becomes viscous, (ii) a nozzle through which the molten material is extruded with controlled speed and diameter, (iii) a build platform which serves as the foundation for the printed object, and (iv) a movable printing unit that generate layer-by-layer deposition of the molten material, as shown in Figure 4c [9]. The typical procedure for printing graphene/polymer composites involves extrusion and deposition, as shown in Figure 4d [75]. The nozzle diameter (*D*) is typically larger than the height (*h*) of the deposited filament, ensuring robust interfacial cohesion among the printed filaments. The ratio of *h*/*D* governs the fundamental configuration of the printed filament, while the ratio of printing head velocity (*v*) and feeding rate (*U*), denoted as *v*/*U*, gives the compression force which leads to intricate geometries of the deposition filaments [78]. During extrusion fabricating of filament, graphene sheets undergo shear force-induced alignment, typically aligning parallel to the screw’s rotational direction and resulting in the filament adopting a vortex configuration [79]. In the subsequent deposition of the filament during 3D printing, the alignment of graphene sheets is influenced by the compression effect with substrate or lower layer, which is related to the layer height parameter [78]. A larger layer height entails diminished compression force, rendering the flat-aligned structure less conspicuous at the upper segment of the filament. Conversely, when the layer height is lower, the compression effect is stronger leading to a more apparent flat-aligned structure at the top and vortex-aligned structures at the filament bottom. The FDM process involves horizontal moving of the nozzle tip during the printing of both flat and vertical samples (Figure 4e) [76,80]. The FDM-printed 3D objects show alignment of deposited filaments, parallel to the printing direction, as shown in Figure 4e,f [76]. The deposited filament layers are interconnected during the cooling and modeling of molten material [81].

The layer-by-layer deposition of fused filaments during the FDM process contributes to a stepwise build-up that is visually evident on the object’s surface. The juxtaposition of these distinct layers creates a distinct, textured, and rough appearance, referred to as the “staircase effect” [82]. This effect manifests as a series of discrete and stepped layers on the vertical surfaces of the printed object, as shown in Figure 4g [76]. The layer height, or vertical resolution, is highly correlated to the final object’s surface finish. Smaller layer heights generally yield finer surface details and smoother finishes, while larger layer heights may result in more noticeable ridges between layers [83]. More detailed morphology information of FDM printed objects are shown in Figure 4g,h [76]. The aligned layers of the deposited filaments are clearly exhibited, together with alignment of graphene sheets in the polymer filaments along the printing direction. The flowing of fused filaments and the movement of the printing nozzle introduced a significant shear effect, attributing to the alignment of graphene sheets.

In FDM, the fabrication process involves the extrusion of molten polymer with dispersed graphene, which is then directly cooled to solidify the material. This direct cooling enables the formation of solid structures without the need for solvent evaporation or post-printing curing processes. Therefore, complex structures with good mechanical strengths are printed using the FDM method, as shown in Figure 4i. Standing words of “FUDAN UNI” and “GRAPHENE” were printed through a FDM nozzle with a diameter of 0.4 mm using graphene/ABS filaments [77]. Small components for electronics and mechanical equipment, such as gear wheels and radiator fans, were also FDM printed [68,76]. Thomas printed a graphene nanoplatelet composite microsatellite assembly, which had a significant weight saving of 60% over that of aluminum alloys [72].

### 2.2. Photopolymerization Strategies

#### 2.2.1. Stereolithography (SLA)

SLA, developed by Charles Hull in the 1980s, is an additive manufacturing technique that uses a process of photopolymerization to create three-dimensional objects. In the usual SLA process, the photopolymerizable liquid resins is meticulously cured with the exposure of UV laser [84]. Numerous layers of this crosslinked and solidified photopolymer are incrementally fabricated, stacking atop one another, until the desired final geometry is achieved. SLA-printed products show a smooth surface finish and high resolution at 20 μm or less, enabling SLA to utilized for rapid prototyping in a wide range of industries such as medicine, aerospace, electronics, and energy storage [85,86,87].

**Figure 5:**
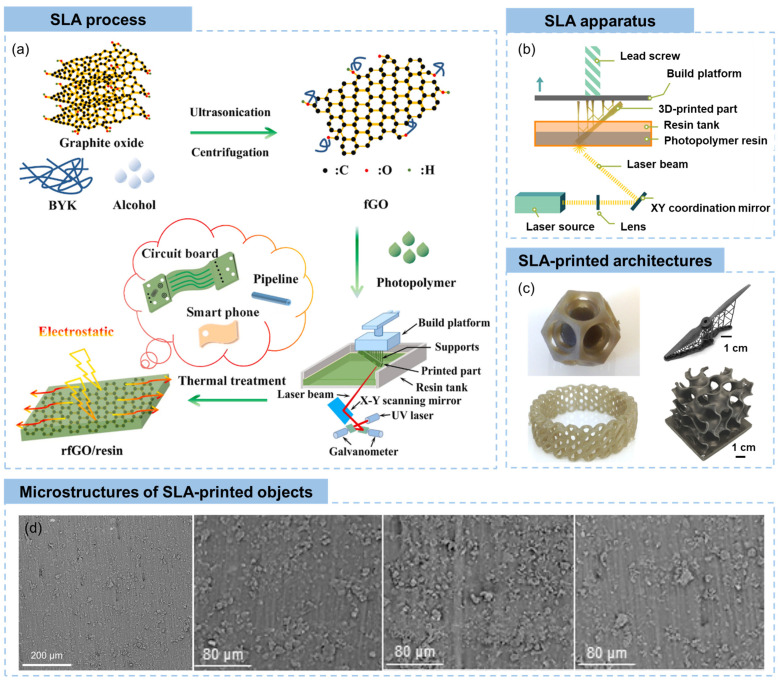
(**a**) A typical fabrication process of functionalized rGO/resin composites by SLA [88], (**b**) Schematic illustration of SLA apparatus [10], (**c**) typical graphene/polymer composite objects printed using SLA [10,93,94], and (**d**) microscopic morphologies of SLA-printed graphene nanocomposites [93].

In a typical fabrication process of reduced graphene oxide (rGO)/resin architectures using SLA (Figure 5a) [88], GO was firstly dispersed and functionalized in solvents, followed by the addition of photopolymer to form a photocurable slurry. The mixture was then UV-polymerized through an SLA printer with controlled layer thickness, wavelength and energy dose of the UV laser. After immersing in solvents to remove excess resins and thermal treatment for post-curing, the resultant SLA-printed component was obtained. The first work on SLA-printed GO/resin nanocomposites was published in 2015, covering the enhanced strength and ductility by 62 and 12.8%, respectively, with a mall filler content of 0.2 wt% [89]. With rational design of GO functionalization by NH_2_/NH_3_^+^ groups, the SLA-printed GO/resin architecture exhibited significant increase in the glass transition temperature and an increment of tensile strength by ~673.6% with an ultralow GO loading of 0.01 wt% [84]. The performances of SLA-printed structures are highly related to the dispersion of graphene precursors, the interfacial interactions of graphene derivatives and polymers, the reduction of GO, and post-curing [84,90,91,92].

The apparatus of SLA mainly consists of a laser source that generate UV lasers for polymerization of photopolymers, a lens and an XY coordination mirror system to control the moving of the UV laser, a build platform that can move up and down to accumulate photopolymerized composites layer by layer, as shown in Figure 5b [10]. Based on the controllable and agile movement of the UV laser, spatially complicated 3D structures can be constructed by SLA (Figure 5c), diverging from the confines of in-plane configurations associated with extrusion-based printing. Both graphene/polymer composites-based structural components [95] and flexible functional prototypes [88,96] can be fabricated by the SLA technique, depending on the choice of the photopolymer. For example, complex-shaped GO nanocomposites of nested dodecahedron and diagrid ring with good mechanical properties were printed by SLA the technique [10]. Palaganas et al. [93] reported a full-scale drone blade fabricated by SLA printing of GO nanocomposite precursor, showing light weight and multifunctional properties, such as improved mechanical and thermal properties. 3D scaffold architecture for biomechanical applications, such as a jawbone with a square architecture and a sternum with a round architecture, were fabricated using SLA, which enables the bone scaffold to be individually personalized and available for clinic applications [94].

Different from the extrusion-based techniques discussed before, SLA-printed objects generally show relatively layerless and continuous build. SLA is a form of vat photopolymerization, where a liquid photopolymer resin is selectively cured by a focused UV laser to solidify it into the desired shape. This process results in objects with smooth and homogeneous surfaces (Figure 5c,d), lacking the visible layering found in other 3D printing techniques [10,93,94]. The near-solid structure with a small percentage of microvoids is a natural outcome of the SLA printing process, as shown in Figure 5d [93]. With increased graphene content, the microvoids are more obvious. Owing to the remarkable UV light absorbance of graphene, the inclined and horizontally oriented graphene sheets obstruct the passage of laser light through the resin directly above them, thereby hindering polymerization in these specific regions and resulting in the formation of microvoids [91].

#### 2.2.2. Digital Light Processing (DLP)

**Figure 6:**
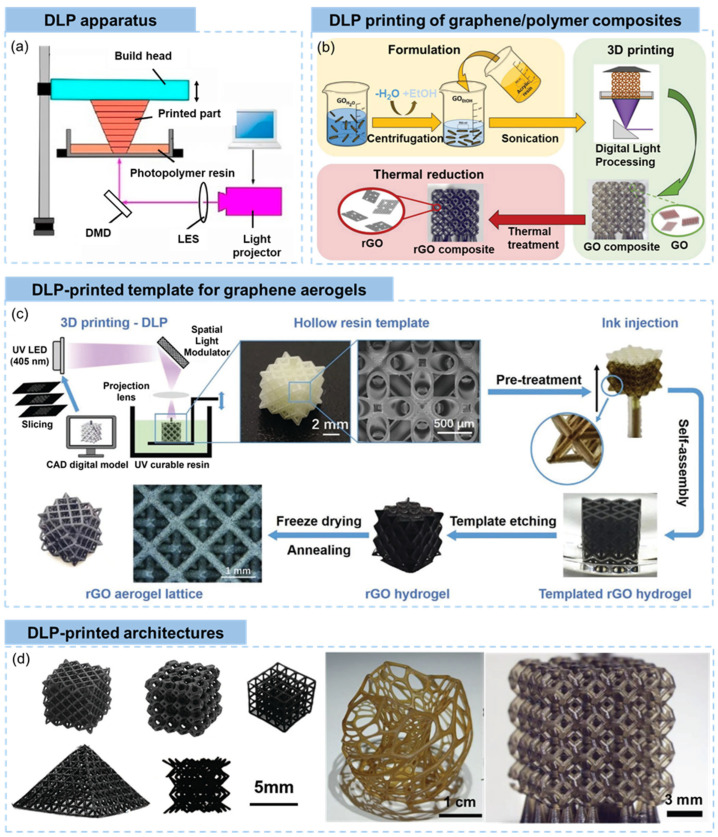
(**a**) Schematic illustration of DLP apparatus [11], (**b**) Typical fabrication process of graphene/polymer composite by DLP printing [102], and (**c**) the fabrication process of graphene aerogel lattice based on DLP-printed hollow structures [103], (**d**) Typical graphene and graphene/polymer architectures printed by DLP [102,103].

DLP utilizes a digital micromirror device (DMD) and photopolymer resins to create three-dimensional objects, which shares similarities with SLA in terms of using photopolymerization to solidify liquid resin [97]. However, the key difference lies in the method of light projection. The schematic illustration of DLP apparatus is shown in Figure 6a [11]. In DLP, the build platform is positioned beneath a liquid resin vat. A DMD, which consists of an array of microscopic mirrors, is used to project UV light onto the resin surface [98]. Each mirror can be individually controlled to direct light either toward the resin or away from it, allowing for the precise control of light patterns and exposure across the entire resin surface. The DLP process of graphene/polymer composites involves the following steps: (i) preparing the 3D model and slicing the 3D model into thin cross-sectional layers, (ii) preparing photocurable graphene/polymer slurries which are typically transparent or translucent to allow UV light penetration, (iii) UV light projection during which the DMD reflects UV light onto the immersed building platform, allowing for the simultaneous curing of an entire layer, (iv) layer-by-layer building achieved by incrementally raised building platform or lowered resin van after the previous UV exposure to accommodate the next layer, and (v) post-processing by rinsing the object in solvents to remove residual uncured resin and further curing [99,100,101]. DLP offers advantages such as faster printing speeds compared to SLA, since an entire layer is cured in a single exposure. It also allows for high-resolution prints with fine details. DLP is commonly used in applications such as rapid prototyping, dental applications, jewelry design, and creating highly detailed models or art pieces.

In addition to the DLP printing system, the graphene/photopolymer is critical to the final DLP printed objects [104]. The general requirements of resins for DLP are (i) suitable viscosity to enable proper coating of the resin onto the building platform, (ii) good stability to prevent settling or sedimentation of solid particles, (iii) well-controlled particle size and uniform distribution to guarantee consistent layer formation, (iv) low shrinkage to maintain dimensional stability, (v) transparency to UV light to allow the UV light to penetrate and cure the resin during the printing process, and (vi) curing efficiency to enable efficient curing of the photopolymer resin using the DLP system’s UV light source [101,105,106,107].

By taking advantage of the solubility of GO in ethanol as well as the miscibility of ethanol and acrylic resins, a concentrated GO liquid crystal ethanol solution with sufficient viscosity was prepared by Tilve-Martinez and coworkers for DLP printing, as shown in Figure 6b [102]. Partially reduced GO was added into the elastomer resin and sonicated to ensure uniform and stable dispersion [108]. Surface modification of as purchased multi-layer graphene nanoplatelets was conducted to ensure the uniformity and stability of the slurry [109,110]. In addition to the fabrication of graphene/polymer composites, the DLP-based 3D printing can also be applied to prepare neat graphene structures. As is shown in Figure 6c [103], DLP printing was used to produce polyacrylate resin templates consisting of different arrangements of interconnected hollow struts, followed by injecting GO suspensions into the space in the centers of the strut. After gelation and self-assembly by hydrothermal process, the template was removed to obtain rGO hydrogels. Graphene aerogel lattices with different complex arrangements of skeletons were fabricated by freeze drying and thermal annealing.

Sharing similar printing mechanisms with SLA (despite the different UV light sources), DLP printing selectively cure an entire layer of liquid resin on the latest cured layer. As a result, the transitions between layers are generally smoother and less pronounced compared to methods that build objects through the incremental deposition of material, such as DIW and FDM. The continuous and smooth surfaces of DLP-printed objects are clearly shown in Figure 6d [102,103]. In terms of microstructures of the printed skeletons, the DLP printed objects show a near-solid structure with the existence of microvoids induced by the UV light absorbance by graphene sheets [111], which is similar to those in SLA-printed materials. Thanks to the photopolymerization-induced 3D printing, the DLP technique can fabricate complex objects with vertical or vertically inclined filaments in straight or curved form (Figure 6d).

### 2.3. Powder-Based Methods

**Figure 7:**
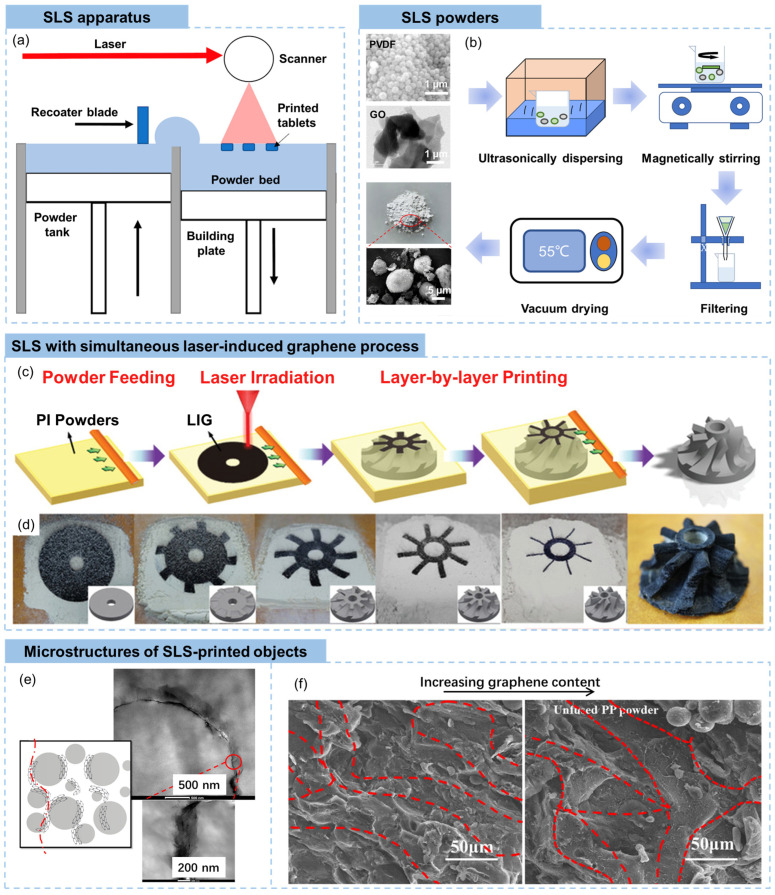
(**a**) Schematic illustration of apparatus [12], (**b**) Typical fabrication process of graphene/polymer powders for SLS-based 3D printing [115], (**c**,**d**) SLS integrated with simultaneous LIG for 3D printing of neat graphene objects [116], (**e**) Transmission electron microscopic [117] and (**f**) scanning electron microscopic images [118] showing microstructures of SLS printed graphene/polymer composites.

The SLS is a typical powder-based additive manufacturing process that is suitable for the fabrication of graphene/polymer composites. Selective laser melting is another commonly used powder-based 3D printing, while it is more applicable for metallic materials [112]. In the SLS, a high-powered laser is applied to selectively sinter powdered materials, typically thermoplastics, to create three-dimensional objects [113]. The SLS apparatus consists of several key components, such as a laser generator, a powder bed for selective sintering, a powder tank for complementary powder supply, a recoater blade for spreading a new layer of powder over the previous layer, and a scanner for SLS, as shown in Figure 7a [12]. During the SLS process, a thin layer of powdered material, known as the building material, is spread uniformly onto a build platform. After a layer is selectively sintered by laser, the build platform is lowered and the powder tank is raising to provide a new uniformly covered layer over the previous layer, followed by selectively sintering the new layer onto the solidified layers beneath. This layer-by-layer building process is repeated until the entire object is formed. In SLS, the unfused powder acts as a natural support structure for the object being printed. It provides stability during the printing process, especially for overhanging features or complex geometries [114]. No additional support structures are typically needed, as the loose powder supports the object during printing. Once the SLS process is finished, the printed object is carefully extracted from the loose powder to remove excess powders by brushing or blowing. Depending on the specific material, additional post-processing steps may be required, such as heat treatment, surface finishing, or infiltration with polymers or metals to improve the strength or surface characteristics.

As described, the SLS is based on the manufacturing of powders; therefore, the quality of the building powders directly influences the final 3D printed objects. Spherical polyvinylidene fluoride (PVDF) powders (average diameter of 200 nm) and GO powders (average diameter of 0.5–3 μm and average thickness of 0.55–1.2 nm) were blended in ethanol which can disperse GO sheets but do not dissolve PVDF powders [115]. After ultrasonication and mechanical stirring, the resultant suspension was filtrated using a funnel to obtain uniform GO-coated PVDF composite powders for SLS, as shown in Figure 7b [115]. Graphene/carbon black/polymer composite powders were also prepared using similar ultrasonic dispersion-assisted liquid deposition, where graphene nanosheets and carbon black were dispersed in organic solvents using ultrasonication before the addition of polymer powders [118,119]. More environmentally friendly aqueous systems were used for in situ reduction of GO sheets with the existence of polymer powders, obtaining rGO-coated composites powders [120]. As graphene-coated polymer spherical powders are applied in the SLS process, the graphene mainly locates among interfaces of the polymer granules, providing electrically conductive percolations and mechanical reinforcements [117,118,119,121]. Solid fabrication of graphene/polymer SLS powders without involving solvents is also applicable. Song et al. [122] milled graphene nanosheets and PVDF pellets in a solid-state shear milling equipment for 15 cycles to ensure uniform dispersion of graphene in PVDF. The composite powder was then extruded at elevated temperatures by a single-screw extruder, followed by cryogenic grinding, drying and sieving to obtain the final composite powder that was suitable for the SLS process.

The SLS was integrated with the laser-induced graphene (LIG) process to print neat graphene objects. The LIG is a 3D porous material prepared by direct lase writing with a CO_2_ laser on carbon-containing material, such as polyimide (PI), cloth, paper, and food, in ambient atmosphere [123,124]. A LIG based additive manufacturing protocol was developed by Liu et al. [116]. On the basis of SLS, they exposed a PI powder bed to UV irradiation, which caused both particle sintering and graphene conversion processes on a layer-by-layer basis. This approach allowed for the assembly of complex graphene architectures, as visually illustrated in Figure 7c.

The morphology of graphene nanocomposites fabricated through SLS is highly diverse, intricately influenced by factors such as powder characteristics, sintering parameters, and post-processing treatments. However, similarly, within the SLS procedure, where layers of material are meticulously fused by a precision laser, the graphene sheet-coated polymer powders coalesce to unveil a distinctive segregated distribution [125]. As shown in Figure 7e, the transmission electron microscopic images of SLS-printed graphene/thermoplastic polyurethane (TPU) composites clearly demonstrated that the surfaces of TPU particles was covered by graphene sheets, forming a 3D segregated network [117]. Shen et al. [118] revealed the effect of graphene content on the segregated distribution. With a larger graphene content, the distance between adjacent polypropylene particles increased, showing wider graphene-rich regions (Figure 7f). A decrease in the sintering of the polypropylene powder at a higher graphene content, visualized by unmelt polymer particles, is shown in Figure 7f.

### 2.4. Post-Processing

While each section of 3D printing techniques briefly touched upon the post-processing of 3D printed graphene and graphene/polymer composites, this section aims to offer a more comprehensive exploration of the pivotal post-processing steps. It is recognized that diverse 3D printing methods demand distinct post-processing approaches to optimize material properties and end-product quality. Recognizing the unique attributes of each 3D printing technique, this section will delve into the tailored post-processing strategies for each method.

Upon completion of the DIW printing process, the removal of residual solvents from the printed objects becomes imperative. To achieve this, three primary strategies have been developed, namely freeze drying, supercritical fluid drying, and ambient-temperature drying, all aiming to preserve structural integrity while facilitating effective drying [126]. Freeze drying and supercritical fluid drying as post-processing methods yield graphene-based aerogels with intricately defined structures, whereas ambient-temperature drying may induce structural shrinkage to some extent [43,126,127]. To reinstate the graphitic structure and exploit the remarkable attributes of graphene, chemical or thermal reduction is often essential for printed objects containing GO building blocks. [126]. Additionally, depositing functional materials onto the high-specific-area 3D DIW printed graphene aerogels has been proved to be effective in imparting specific functionalities to the final aerogels. For example, Yao et al. engineered MnO_2_/rGO aerogel lattices via post-electrodeposition of MnO_2_ nanosheets, thereby conferring exceptional supercapacitive performances upon the printed lattice [128].

The characteristic “staircase effect” in FDM printed materials is readily discernible, presenting a distinct layer-by-layer morphology. To address this, thermal annealing emerges as a critical intervention, facilitating an advanced level of fusion and bonding amidst the sequentially deposited layers [129]. This transformative process significantly fortifies the structural integrity, concurrently ameliorating the porosity and mechanical properties of the printed structure. In scenarios involving composites featuring specific thermoplastic constituents, such as ABS, an innovative technique known as acetone vapor smoothing holds paramount importance [130]. This procedure involves subjecting the printed object to acetone vapor, inducing localized surface melting followed by re-solidification of the outer layer. Subsequent to the application of solvent vapor smoothing, the formerly semi-circular surface layers of the FDM printed objects undergo a remarkable transformation, attaining a newfound level of smoothness and coherence.

Comparable post-processing steps are requisite subsequent to SLA and DLP processes which both employ the solidification of liquid resin layer by layer via UV exposure. Upon the completion of printing, the object typically undergoes immersion in a solvent bath, which is essential to eradicate surplus uncured resin [99]. This strategic immersion mitigates tackiness and ensures a pristine surface quality. While UV light exposure is inherent to SLA and DLP printing during the fabrication stage, supplementary UV irradiation may be employed to attain comprehensive curing and reinforce the mechanical properties of the printed material [110]. Notably, if support structures are integral to certain SLA and DLP printed objects to provide stability during fabrication, subsequent elimination of these supports necessitates meticulous attention. Methods such as cutting and sanding are adroitly employed to skillfully detach these support structures, guaranteeing an unblemished final outcome.

Upon the completion of the SLS process, the printed object is carefully extracted from the surrounding loose powder, followed by elimination of surplus powders by either precise brushing or controlled blowing [120]. As discussed in the section of “Powder-based methods”, microvoids exist in SLS printed materials. Therefore, the critical post-processing of SLS fabricated materials is thermal annealing [131]. Controlled heat treatment is often applied to further enhance the bonding between the sintered particles, promoting increased density, improved mechanical strength, and reduced porosity. This step is particularly effective in achieving higher material integrity and stability.

### 2.5. Comparisons

Based on the above review on 3D printing techniques for graphene and graphene/polymer composite, comparisons of the above-discussed five methods are shown in Table 1. Among them, the FDM, SLA, DLP, and SLS are not applicable for direct printing of neat graphene or GO because of the involving of melting, photocuring or sintering process, while sacrificing templates for graphene macroscopic assemblies can be printed followed by graphene infiltration and template etching [103]. For the printing of graphene/polymer composites, all the five methods are applicable. Viscous ink (for DIW), continuous filament (for FDM), and photocurable polymer-based slurry (for SLA and DLP) precursors for 3D printing generally result in uniformly dispersed graphene sheets in polymer matrices, while the graphene-coated polymer powders used in SLS generate segregated graphene [132,133]. On the basis of the molding method, the extrusion-based DIW and FDM are effective in printing in-plane frameworks, while showing limitations in vertical printing. As a comparison, the spatial complexity of printed objects by SLA, DLP, and SLS is more desirable, enabling designing and fabrication of components with complex structures. Among the five mentioned techniques, DLP shows the highest efficiency since an entire layer can be printed upon single exposure of UV light.

## 3. Multifunctional Applications of 3D Printed Graphene and Graphene/Polymer Composites

### 3.1. Energy Storage

#### 3.1.1. Supercapacitors

Supercapacitors, also known as ultracapacitors or electrochemical capacitors, are a type of energy storage device that store and release electrical energy quickly and efficiently. They operate on the basis of electrostatic charge storage within the electrochemical double layer at the electrode–electrolyte interface, offering high power density, a long cycle life, and quick charging. This makes them suitable for various applications such as electric vehicles, renewable energy systems, and consumer electronics [134]. Therefore, graphene and 3D printed graphene-based structures with a large specific surface area, high electrical conductivity, and excellent structural stability are highly suitable for application in supercapacitors.

**Figure 8:**
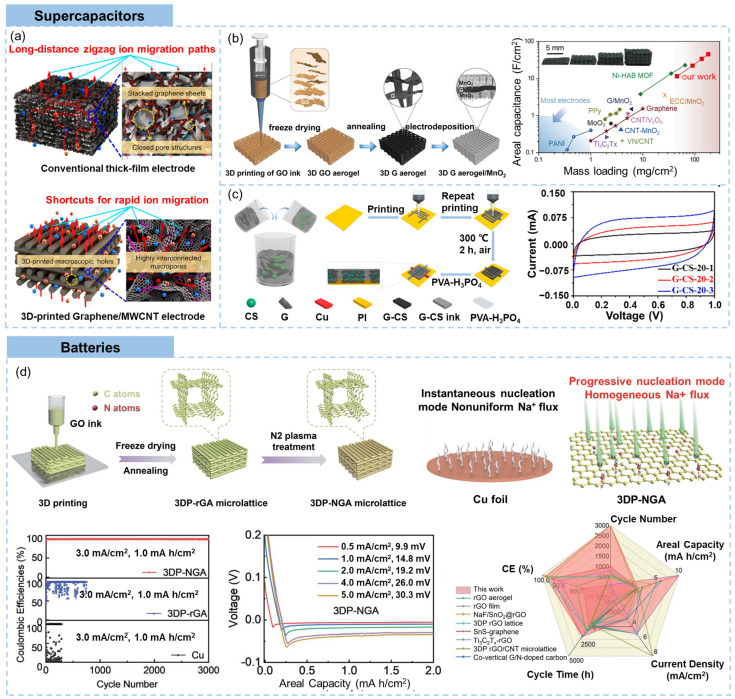
3D printed graphene supercapacitors: (**a**) schematic illustrations of insufficient ion transport in a conventional thick-film electrode and sufficient ion transport in a 3D-printed Graphene-based aerogel electrode [39], (**b**) MnO_2_-incorporated 3D printed graphene supercapacitors [128], (**c**) 3D printed in-plane interdigital structured microsupercapacitors using graphene/carbon sphere inks [138], (**d**) 3D printed graphene batteries [139].

The 3D printed graphene structures show superiority over traditional thick-film electrodes in terms of supercapacity, as shown in Figure 8a [39]. In conventional thick-film electrodes, the tortuous and irregular networks often result in enclosed pore structures, rendering them impermeable to electrolyte infiltration and causing inefficient utilization of active materials. Moreover, ions have to traverse elongated routes along zigzagging pathways generated by stacked graphene sheets within the film, causing a low diffusion speed [135]. In contrast, the interconnected hierarchical pores of 3D-printed structures construct unimpeded conduits for ion movement, spanning from the bottom to the top surface. The ample junctions between adjoining filaments and percolated graphene networks within filaments forge sufficient pathways for electron conduction [99,136,137]. The structural stability of 3D printed freestanding electrodes that can avoid permanent deformation adds to the excellent areal performances of 3D printed graphene supercapacitors [39].

Many efforts have been made to optimize the supercapacitance of 3D printed graphene [99,140,141]. To ensure the large specific surface area of the 3D printed structure, freeze drying or activation processes are generally conducted after printing, which ensures a large-area interface between the hierarchical porous graphene electrode and the electrolyte [142,143,144]. Zhu et al. reported 3D printed aerogel microlattices using composite inks consisting of GO, hydrophilic fumed silica powder, graphene nanoplatelet, and a catalyst [145]. The supercapacitors assembled using these 3D-aerogel electrodes with thicknesses on the order of millimeters display exceptional capacitive retention (ca. 90% from 0.5 to 10 A/g) and power densities (>4 kW/kg).

The supercapacitive properties of 3D printed graphene aerogels were further enhanced by surface functionalization of graphene sheets using electrochemical oxidation in 0.5 M KNO_3_ solution at a potential of 1.9 V versus saturated calomel electrode [146]. With an open structure that ensured sufficient functional groups on carbon surfaces and that facilitated the ion accessibility to these functional groups even at high current densities, an asymmetric device exhibited a remarkable energy density of 0.65 mW h/cm^2^ at an ultrahigh power density of 164.5 mW/cm^2^. A graphene aerogel microlattice fabricated by Ca^2+^ crosslinking-assisted DIW exhibited wrinkled porous structure and coherent conductive network, showing gravimetric capacitance of 213 F/g at 0.5 A/g and 183 F/g at 100 A/g, as well as a retention over 90% after 50,000 cycles [33]. A 3D-printed graphene aerogel electrode loaded with MnO_2_ was developed by DIW-based printing of GO ink followed by freeze drying, thermal annealing and electrodeposition of MnO_2_ nanosheets, as shown in Figure 8b [128]. The high mass loading of MnO_2_ (182.2 mg/cm^2^) on the 3D printed graphene aerogel electrode yielded high areal capacitance (44.13 F/cm^2^, a superior electrochemical performance compared to electrodes with other carbon substrates under similar loading conditions), excellent conductivity, and efficient ion diffusion. Through the symmetric arrangement of two 4 mm-thick electrodes the resultant device exhibited a notable energy density of 1.56 mW h/cm^2^. Moreover, this electrode, comprising a 3D-printed MnO_2_-deposited graphene aerogel, achieved exceptional capacitance values normalized to area, mass, and volume simultaneously, overcoming the typical trade-offs associated with most electrode materials.

In-plane microsupercapacitors with a compact size are a type of microscale energy storage device designed to be integrated into electronic circuits or systems. The addition of spacers of carbon sphere and a self-sacrificing template of ethyl cellulose in graphene inks enabled the 3D printing process of a graphene-based interdigital-structured microsupercapacitors, as shown in Figure 8c [138]. The corresponding assembled interdigital-structured microsupercapacitors with a lateral size of several millimeters and a thickness of 120 μm delivered high areal capacitance (30 mF/cm^2^), high energy density (4.17 μW h/cm^2^) and power density (0.22 mW/cm^2^). When printed on a flexible substrate, flexible supercapacitors are fabricated [147,148].

#### 3.1.2. Batteries

3D printing for graphene-based batteries represents an innovative approach to fabricate advanced energy storage devices that harness the unique properties of graphene [149]. Graphene, with its high electrical conductivity, large surface area, and excellent mechanical strength, offers significant potential for improving battery performances. It opens up opportunities for creating high-performance, lightweight, and customizable batteries that can find applications in consumer electronics, electric vehicles, aerospace, and renewable energy systems. As research and developments continue to progress, 3D printed graphene-based batteries are poised to revolutionize the energy storage landscape with their unique capabilities and versatility.

3D printing allows for intricate and complex battery designs, which can be tailored to specific applications. 3D printed GO framework with thermal shock synthesized nanoparticles for Li-CO_2_ batteries [150] and holy GO 3D architectures on arbitrary substrate for Li-O_2_ batteries [151] are typical examples to utilize the structural designability of additive manufacturing for enhanced energy storage capacities. The high surface area of 3D graphene structures provides more reaction sites and also allows for enhanced ion diffusion and improved accessibility to active sites, leading to higher energy storage capacity and faster charge/discharge rates [152]. 3D printing enables the integration of multiple battery components (such as electrodes, current collectors, and separators) into a single, monolithic structure, simplifying assembly and reducing manufacturing steps [153]. An all-component 3D-printed lithium-ion batteries was fabricated using GO-based composite inks (consisting of highly concentrated GO sheets along with cathode and anode active materials) and solid-state electrolyte inks [154]. A meticulous layer-by-layer deposition of fine filaments constructed an interdigitated battery configuration. The resultant fully 3D-printed battery demonstrated a notable electrode mass loading of approximately 18 mg/cm^2^ when considering the total battery area. This configuration yielded initial charge and discharge capacities of 117 and 91 mA h/g, respectively, while maintaining commendable cycling stability.

Another contribution of the 3D porous graphene-based architecture is its ability to inhibit the growth of metallic dendrites during charge and discharge cycling. As shown in Figure 8d [139], a 3D-printed N-doped graphene microlattice aerogel (3DP-NGA) for sodium metal anode was fabricated by DIW printing of GO inks followed by freeze drying and N_2_ plasma treatment. The 3D porous anode structure with high surface areas decreased the local current density and inhibited the growth of sodium dendrites. The N doping through controllable plasma treatment reduced the nucleation potential and promoted uniform sodium ion flux, which make additional contributions to the uniform deposition of sodium metal (the schematics in Figure 8d). As a result, the 3DP-NGA delivered an average coulombic efficiency of 99.90% at a high current density of 3.0 mA/cm^2^ with 1.0 mA h/cm^2^. The 3D-printed full battery achieved a capacity of 85.3 mA h/g at 100 mA/g after 1000 cycles, which outperformed most graphene-based sodium metal batteries. The incorporation of carbon nanotubes (CNTs) into the 3D printed graphene microlattices was also beneficial to reduce the local current density and enhance the uniform nucleation and deposition of sodium metals [155]. Similar inhabitation of the 3D-printed porous architecture in metal dendrite growth has been confirmed in zinc ion batteries [156].

### 3.2. Sensing

#### 3.2.1. Strain Sensing

Working as substantial intermediates for collecting external mechanical signals, flexible strain sensors are regarded as indispensable components in flexible integrated electronic systems. Graphene-based strain sensors is an emerging and promising application that leverages the exceptional electrical properties of graphene to create highly sensitive and customizable sensors for measuring mechanical deformation or strain. The 3D printing technique has recently been used to fabricate flexible sensors due to its high accuracy, simplicity, and rapid prototyping [157,158]. GO inks were directly wrote on polyurethane polyurethane (PU) substrates, followed by freeze drying and reduction, to achieve deformation-sensitive resistances for strain sensing [159,160]. Qian et al. reported a flexible strain sensor based on rGO/elastomer resin composites printed by DLP, showing a sensitivity of 6.7 at a linear strain response range of 0.01–40% and a high mechanical stability of beyond 10,000 stretching/releasing cycles [108]. The parameters (such as included angle between printed filaments, width and thickness of skeletons) and the printable materials (such as filler content and dispersion) of printed structure are highly related to their strain-sensing properties [161,162,163]. The SLS-fabricated composites based on graphene-coated TPU composite powders created percolated segregated graphene networks [125]. Upon stretching, the percolated graphene network was partially broken, leading to increased resistance with tension. This 0.2 wt% graphene reinforced composites reached a gauge factor of 668.3 at a tensile strain of 15%. SLS integrated laser-induced graphene with a low impedance of <100 Ω, high crystallinity, mechanical robustness and flexibility, and durability exhibited its superiority in sensing electrophysiological signals [164].

Hybrid filler are applied to construct the conductive networks for optimization of 3D printed graphene-based strain sensor. FDM-printed high-performance flexible strain sensors using CNT and graphene nanoplatelet-filled TPU composite filaments showed a high sensitivity (gauge factor = 136,327.4 at 250% strain), a wide detectable strain range (0–250%) and good stability [165]. In addition to the enhancement in strain sensing, the addition of CNT reduced the temperature coefficient of resistance of graphene/polymer composites, obtaining a near-zero temperature coefficient of resistance for DIW-printed graphene/CNT/polymer fibers [166]. With problem of temperature disturbance overcame, this strain sensor exhibited a high sensitivity (gauge factor = 14,550.2 at 100% strain), a wide working range (1–100%), a quick response time (170 ms) and a high durability (10,000 loops). DLP-manufactured graphene/CNT/TPU composites for high-speed complex strain sensors were reported by Liu et al. [167], providing ideas for rapid fabrication of personalized customized wearable electronic devices.

**Figure 9:**
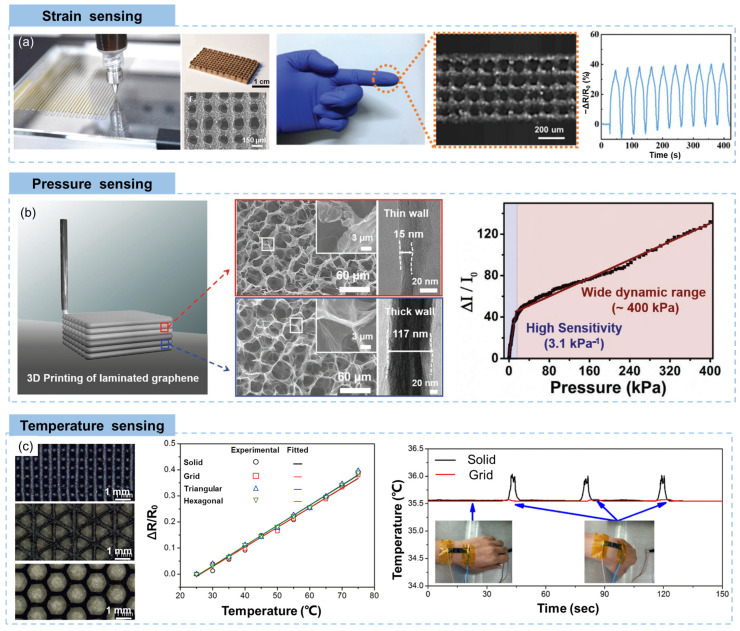
(**a**) 3D printed multilayer GO aerogel lattices and their miniature tape sensor for strain sensing [168], (**b**) 3D printed graphene pressure sensors [169], and (**c**) 3D printed graphene/PDMS composites for strain-insensitive temperature sensing [53].

The printing resolution of additive manufacturing plays an important role in determining performances of 3D printed strain sensors. A higher printing resolution results in more functional units exist per unit area, which can accurately sense deformations of objects (such as robot hand) in the form of resistance changes. Chen et al. [168] developed a printable GO ink which was formulated though modulating oxygen functional groups for ultrahigh-resolution (70 µm) printing. The high quality and low concentration of the GO ink enabled ultraprecise printing with well-defined frameworks in both top and side views as well as a regularly assembled multi-layer structure (Figure 9a). Benefiting from the printing resolution of 70 µm, this rGO aerogel lattice showed better performance and data readability when applied as microsensors and robot e-skin.

#### 3.2.2. Pressure Sensing

The combination of artificial intelligence and robotics has become one of the fast-growing research fields and is considered to have a profound impact on human soci-ety. The development of robotic skin capable of perceiving external pressure environments, or the pressure sensing, is highly desirable for intelligent robots. Besides, pressure sensors find extensive applications in various industries, including automotive, aerospace, medi-cal, industrial automation, consumer electronics, and environmental monitoring. Based on the electrically conductive nature of graphene sheets, the graphene or gra-phene/polymer composite pressure sensors are generally piezoresistive, which directly convert the external pressure to an electrical signal (resistance, current, or voltage change) [170,171,172]. The pressure sensing mechanism is that the change in contact areas/points of conductive graphene under external compression loading significantly determines elec-trical properties of graphene-based materials [173,174]. 

Taking advantages of the flexibility of structural design by 3D printing, different conductive networks (such as square, hexagonal, circular pores, planarly filamented) can be designed, showing different pressure sensing capacities [175,176]. Highly flexible pressure sensors fabricated using 3D printing technique showed improvements in both the sensitivity and the sensing range through the piezoresistive effect [177]. Huang et al. fabricated a graphene-based pressure sensor by 3D printing, showing a nonmonotonic sensing response to compressive stress [163]. A patterned rGO/micro-structured polydi-methylsiloxane (PDMS) sensor with irregular microstructures that were generated in the SLS process achieved a high sensitivity (55 kPa-1) and a wide linearity range (100 kPa) [178]. Inspired by the synergistic effect of dual mechanoreceptors in human skin, a inte-gration of well-designed 3D printing of laminated graphene pressure sensing material consisting of both ultrathin-walled and thick-walled cellular micro-structured layers was developed by Cao et al, as shown in Figure 9b [169]. A carbomer hydrogel-based graphene ink with an ultralow solid content of 3 mg/mL was used for DIW printing of low-modulus thin-walled graphene aerogel layer, while an ink with 35 mg/mL solid content was ap-plied for DIW of the thick-walled layer. Owing to the unique 3D printed structure, this graphene-based piezoresistive pressure sensor achieved a low detection limit (1 Pa), a wide detection range (1 Pa-400 kPa), high sensitivities (3.1 and 0.22 kPa-1 in pressure re-gions of 1 Pa-13 kPa and 13-400 kPa, respectively).

Laser-induced graphene with a layered alignment and porous structure is suitable for pressure sensing. With careful adjustment of the laser power, a laser-induced graphene on-skin sensor for on-body monitoring, robotic hand control, and embedded machine learning for signal classification was designed [164]. 3D printing of polyether ether ketone and double-side laser scribing graphene induced a rationally designed network with a special core-shell structure [179]. Based on the triboelectric effect, this 3D printed structure can serve as self-powered pressure sensors.

#### 3.2.3. Temperature Sensing

3D printing techniques also show great significance in temperature sensing [180,181,182]. For example, a fiber-shaped temperature sensor was fabricated by twisting together the 3D-printed functional fibers [183]. The rGO fiber was mainly responsible for temperature sensing due to the thermal activation that led to the generation of electron and hole pairs, giving a temperature sensitivity of 1.95% °C^−1^. Extrusion-printed CNT/graphene lines using hybrid inks with different ratios of CNT and graphene exhibited temperature-dependent resistance with a significantly large negative temperature coefficient of resistance, showing an averaged resistance drop (per unit temperature) of −3.5 Ω/°C [184]. A 3D printed graphene film showed advantage over conventional temperature sensors due to its wide temperature range of 10–3000 K and flexibility [185]. A tandem line-type temperature sensor based on rGO was fabricated by the DIW technique, exhibiting a temperature sensitivity of 1.2% °C^−1^, and it can be integrated using all-printed all-in-one configuration composed of a asymmetric microsupercapacitor and the temperature sensor [186].

By constructing complex geometries of graphene/polymer composite by 3D printing, flexible pressure– or strain–temperature dual parameter sensors utilizing independent piezoresistive and thermoelectric sensing mechanisms, respectively, can be achieved. A graphene-coated carbon nanofiber (CNF)/TPU architectures endowed the sensor with a high pressure sensitivity of 0.14 kPa^−1^ (0–60 kPa) and a high temperature sensitivity of 30.8 μV/K [187]. This sensor showed neglectable mutual interference between pressure and temperature signals, because of the lowered temperature-induced resistance change caused by surface coating of graphene and the hierarchical structure. A printed graphene-MXene (a family of 2D transition metal carbides and nitrides) sensor also delivered bimodal sensing properties, showing strain-sensitive but temperature-independent resistances as well as temperature-related direct current Seebeck voltage [188].

Flexible temperature sensors often grapple with the challenge of sensing material deformation, leading to complications in maintaining sensing accuracy and reliability due to external strain-induced resistance fluctuations. Wang and colleagues addressed this issue by utilizing DIW to create graphene/PDMS composites with intricate geometries such as grids, triangles, and hexagons for strain-insensitive temperature sensing [53]. the cellular composites, particularly the grid configuration, exhibited temperature sensitivity akin to their solid counterparts. However, they showcased significantly enhanced temperature sensing stability, characterized by minimal resistance variation in response to external strains, as shown in Figure 9c. This was because that the fine porous structure effectively shared the external strain.

### 3.3. Stretchable Conductor

**Figure 10:**
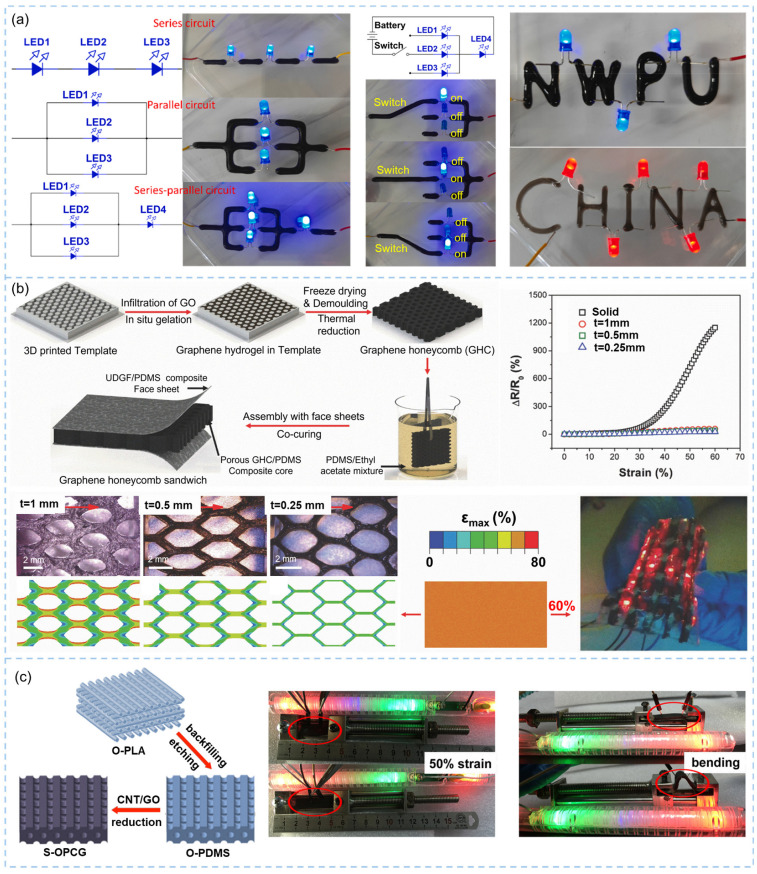
3D printing-based graphene/polymer composites for stretchable conductors: (**a**) customized graphene conductors for different circuits and switches [193], (**b**) hierarchical graphene aerogel/PDMS composites with honeycomb porous structures [197], (**c**) graphene/CNT/PDMS composites with 3D printing-controlled pore structure [198].

The addition of electrically conductive graphene sheets into a polymer matrix is a common strategy to make the intrinsically insulating polymer conductive [189]. To achieve electrical conduction, sufficient connections among conductive fillers to form a percolated conductive network are necessary [190]. 3D printing offers a versatile and innovative approach to creating electrically conductive components, enabling the fabrication of conductive structures with customized geometries and intricate designs that are difficult to achieve using conventional manufacturing methods [64,191,192]. Chen et al. constructed a series circuit, a parallel circuit and a series-parallel circuit by printing the conductive sodium alginate/graphene/CNT hydrogel without glue on the pin of a light-emitting diode (LED), as shown in Figure 10a [193]. Furthermore, this hydrogel was utilized to create a single-pole triple-throw switch, enabling the LED to be illuminated by manipulating the hydrogel switch. Even when the circuits were configured in the shape of the words “NWPU” and “CHINA” (Figure 10a), the normal operation of the system was effectively sustained. Moreover, the 3D printing technique produced lattice structure is capable of sharing external strain, making it a perfect candidate to fabricate 3D graphene with a precisely controlled structure for stretchable conductors [194].

With rational design, DIW printed graphene aerogel lattices can serve as flexible conductors, either stretchable or compressible. Zhu et al. [195] developed a highly compressible 3D periodic graphene aerogel lattice using DIW printing of GO inks, achieving a lightweight density of 123 mg/cm^3^, an electrical conductivity of 278 S/m and a super-compressibility up to 90% compressive strain. DIW printing of electrically conductive graphene aerogel monolith using large-size GO sheets precursors enhanced the compressibility (up to 80% compressive strain), electrical conductivity (41.1 S/m), specific strength (10.7 × 10^3^ N m/kg) and lowered density (12.8 mg/cm^3^) [37]. Ca^2+^ gelled GO/CNT hybrid inks were used for DIW printing of aerogels, followed by pre-buckled reduction, to produce highly stretchable graphene/CNT hybrid aerogels [127]. The highly conductive aerogel with an electrical conductivity up to 1000 S/cm was capable of sustaining long-term repeatable stretching to strains of 100%. Similarity, high-speed flexographic printing using graphene inks can also be used to prepare complex conductive paths for flexible electronics, showing comparable electrical conductivity at flat and bended states [196].

The incorporation of elastomer matrices significantly enhances the mechanical robust, flexibility and stability of printed objects. Robust, flexible and conductive multilayer 3D printed graphene/polycaprolactone composites containing 10 wt% graphene were fabricated by the DIW process [199]. Highly conductive graphene flexible circuits printed by FDM served as excellent flexible conductors [200]. The effects of the arrangement of composite filaments in 3D printed structures on their flexible conducting properties were revealed using DIW printing of graphene/PDMS composites, demonstrating that the grid structure was more stable than the triangular and hexagonal structure in terms of electrical conductivity under deformation [161]. More typical studies on 3D printed graphene/polymer composites are reviewed as follows.

3D graphene monoliths with controlled macrostructures can be fabricated through DIW using GO inks [30], but generally high-concentration inks are necessary. This results in highly agglomerated graphene sheets within pore walls, making them difficult to be infiltrated fully and uniformly by liquid elastic polymers to stretchable composites. As an alternative, 3D printed lattices can be applied as sacrificial templates for constructing ordered porous structures. Wang et al. [197] printed honeycomb polymer pillar template followed by infiltration of GO dispersion for hydrothermal treatment, freeze drying, and demolding, as shown in Figure 10b. The honeycomb-structured conductive polymer composites with different honeycomb wall thicknesses exhibited significantly lower resistance increments upon stretching. The cellular structure remained intact after stretching to 160% of the original length while the inclined walls rotated towards the loading direction. The finite element analysis revealed that the 3D printed hexagonal composite structure rearranged localized strains, and a thinner wall resulted in less strain localization. Attributed to the slight real strains on the graphene skeleton, the composites with a wall thickness of 0.25 mm were promising applied in highly flexible light-emitting display.

Coating graphene nanosheets or graphene-based hybrid conductive materials on 3D-printed porous structure is another effective approach to combine carbon nanomaterial with 3D porous polymer substrates and thus to manufacture high-performance stretchable conductive materials. The eco-friendly and easy-moving PLA was firstly 3D printed to form a designed structure with spacing between neighboring skeletons being 200 μm, followed by backfilling PDMS with the assistant of vacuum and subsequent PLA etching [198]. The porous PDMS skeleton obtained based on 3D printed structure served as substrates for graphene/CNT coating, obtaining the final conductive composite, as shown in Figure 10c. Under uniaxial stretching to 100% strain, the composite showed a 40% retention of conductivity. In addition, stable electrical conductivity after 5000 bending cycles and a light decrease after 100 stretching cycles of 50% strain were achieved, attributing to the de-localized strain induced by the 3D continuous porous structure.

### 3.4. Electromagnetic Interference Shielding (EMI) and Wave Absorption

#### 3.4.1. EMI Shielding

Owing to the fast development of modern electronics, communications, and industries, the spatial environment in earth is constantly exposed to electromagnetic radiations which are hazardous to our health and safe operation of electronic devices. To minimize the negative effects of electromagnetic radiations, electromagnetic interference (EMI) shielding and wave absorbing materials are developed. Mechanisms behind the EMI shielding of a material mainly includes reflection (results of interactions between the electromagnetic waves and free charges on materials’ surfaces), absorption attenuation (energy dissipation and consuming processes such as localized current and polarization/relaxation of dipoles and free charges), and multiple reflection (negligible when absorption-induced shielding effectiveness (SE) is larger than 10 dB) of electromagnetic waves [201,202]. The 3D printing technique that is capable of rapid production of objects with a wide range of structures have shown its promises in fabricating multifunctional EMI shielding materials.

The FDM method is widely used for the fabrication of 3D printed EMI shielding materials [203]. The study on the influence of specimen thickness and internal geometric designs of graphene/polyamide-6 composites on EMI shielding properties revealed that the increased specimen thickness within 1–5 mm did not enhance the EMI SE [204]. The dispersion of graphene plays an important role in determining the EMI shielding properties of 3D printed graphene/polymer composites. A ball milling and screw-extruding were applied for dispersing graphene sheets in FDM filaments [205]. Benefiting from the well-distributed graphene sheets and the FDM-induced unique porous lamellar structure, this composite achieved an EMI SE of ~32.4 dB (with thickness-normalized specific EMI SE of 318 dB cm^2^/g) in the frequency range of 8.2–12.4 GHz. A local enrichment strategy was developed to fabricate graphene/PLA nanocomposites for FDM printing by precisely manipulating the selective distribution of fillers, obtaining segregated graphene sheets in the printed object [206]. The resulted material showed a SE of 34.7 dB at a filler loading of 10 wt%. Shi et al. used a solution-blending method of graphene nanosheets and PLA to guarantee uniform distribution of graphene before extruding of composite filaments which were used for the FDM printing [207]. The corresponding graphene/PLA composites with a filler content of 9.08 vol% exhibited an EMI SE of 34.9 dB at the X-band region, shielding 99.97% of incident electromagnetic waves. Similar fabrication method for graphene/poly(vinyl alcohol) resulted in a SE of 26–32 dB in the frequency range of 8−12.4 GHz, which also met the practical application requirement of 10 dB [208].

**Figure 11:**
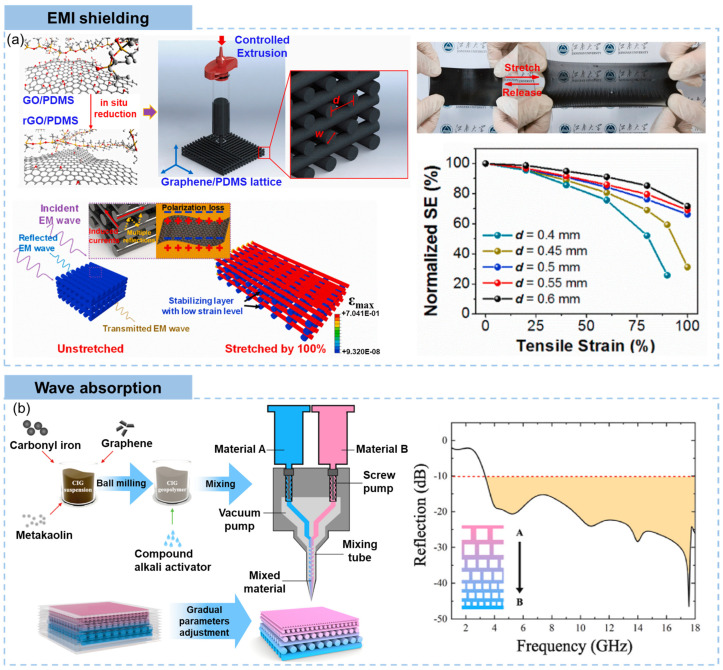
3D printed graphene composites for (**a**) EMI shielding [209] and (**b**) wave absorption [212].

In situ reduction of GO in PDMS solutions followed by viscosity adjustment and DIW printing provided highly stretchable and conductive graphene/PDMS composite lattices, as shown in Figure 11a [209]. Due to their distinctive 3D interconnected and resilient conductive network, the composite lattices displayed remarkable stretchability, reaching up to 130%. They also demonstrated tunable EMI SE of up to 45 dB, coupled with exceptional endurance (maintaining over 90% of EMI SE even after 200 times of cyclic stretching and releasing at strains up to 100%). Impressively, the resulting lattice structure maintained significant shielding stability even when subjected to stretching, retaining over 70% of the original SE when stretched to 100%. This was attributed to the lattice’s ability to distribute external strain effectively, with filaments that were perpendicular to the external load acting as stabilizing layers, thereby mitigating abrupt resistance variations. 3D micropatterned Fe_3_O_4_ functionalized graphene/polymer nanocomposites achieved a high electrical conductivity of ~580 S/m and an EMI SE of over 50 dB in the X-band [45]. When integrated with freeze drying, the DIW printed architectures exhibited hierarchical pores which make additional contributions to the EMI shielding properties of materials [201]. The DIW printed graphene/CNF composites with porous skeletons exhibited an excellent EMI SE as high as 55.6 dB in the X-band region [210]. The architectures that were printed using GO/MXene inks showed a high electrical conductivity of 1013 S/m and a broadband tunable EMI SE of above 60 dB in the frequency range of 8.2–26.5 GHz [211]. Attributed to the ultralow density of 16 mg/cm^3^, the DIW printed and frozen dried scaffold achieved an ultrahigh normalized surface specific SE of up to 19,270 dB cm^2^/g.

#### 3.4.2. Wave Absorption

Wave absorption, also known as electromagnetic wave absorption, is a process by which specific materials or structures absorb and dissipate electromagnetic energy in the form of heat. Different from EMI shielding which is about creating a barrier to prevent electromagnetic waves from entering or escaping a device, wave absorption focuses on dissipating electromagnetic energy as heat to reduce the intensity of the waves and reduce the reflection of incident radiations [213]. Wave absorption is particularly useful in aerospace, military target anti-radar stealth, and environments with multiple electronic devices emitting electromagnetic waves, etc.

Thanks to the flexibility and effectiveness of 3D printing in structural design, multilayer rGO/PLA absorbers were designed with a gradient index of characteristic impedance by adjusting the rGO content and the geometric parameters of the unit cell, with the seven-layer material giving an absorption above 90% in a broad bandwidth of 4.5–40 GHz [214]. A functionally grade geopolymer containing carbonyl-iron and graphene (CIG) powders with graded and porous structures were successfully constructed to enhance the wave-absorbing property via dual gradient DIW-based 3D printing, as shown in Figure 11b [212]. Two inks, namely the material A containing 80 wt% of CIG and the material B of neat geopolymer, were extruded and deposited under well-controlled mixing to create a conductive filler gradient from the bottom to the surface. The structural gradient, that is the rod spacing, was controlled by the printing process. The dual gradient lattice achieved a wave absorption capability of a minimum reflection loss of −46.47 dB at 17.58 GHz and a broadband absorption of 14.62 GHz (3.38–18.00 GHz). The continuously gradient composition contributed to the impedance matching, while the EM interference effects of components contributed to the macroscopic gradient structure design and the intrinsic foaming structures.

There are many other explorations of 3D printing in wave absorption. For example, the DLP printed CIG/poly (methyl methacrylate) (PMMA) nanocomposites with various compositions presented a minimum reflection loss of −54.4 dB at a thickness of 2.1 mm, along with effective absorption bandwidth of 3.41 GHz [110]. Zuo et al. developed a multi-material bilayer absorbing composites consisting of graphene/Li_0.35_Zn_0.3_Fe_2.35_O_4_/PMMA as the matching layer and CIG/PMMA as the absorption layer via multi-material DLP, achieving a minimum reflection loss of −46.1 dB at 4.7 GHz and an effective absorption bandwidth of 3.5 GHz (4.15–5.35 and 14.7–17.0 GHz) [215]. The ball-milled CIG reinforced PLA for FDM printing of nanocomposites resulted in a minimum reflection loss of 50.1 dB and an absorption bandwidth of 6.0 GHz at a sample thickness of 2.2 mm [216]. Ye et al. fabricated a FeSiAl/PLA and FeSiAl-MoS_2_-graphene/PLA double-layer absorber with effectively improved impedance matching using FDM technology [217]. A minimum reflection loss of −52.5 dB at 17.16 GHz and an effective absorption bandwidth of 5.92 GHz (12.08–18 GHz) were achieved by this double-layer composites.

### 3.5. Bio-Applications

The biocompatible graphene is capable of supporting stem cell growth and osteogenic differentiation as well as adsorbing dexamethasone and β-glycerolphosphate (osteogenic inducers) via π–π stacking, making it beneficial bone tissue engineering [218]. Additive manufacturing is able to feasibly place functional bone-repair materials within composite materials, giving them bone repair functionalities. In addition to biomaterials, 3D structures of the scaffold are critical to define scaffold responses, especially shape, microstructure and porosity [115,219]. In addition, 3D printing produces biomimetic structures on the basis of a computed tomographic image obtained from a patient’s damaged or injured body, creating patient-specific customized structure with desired shape and size [220]. A polydopamine-reduced GO reinforced 3D printed PLA scaffold with well-defined porosity for bone tissue construction was reported by Aharma et al. [221], displaying stem cell responsive multi functionalities and exhibiting potentials as bone tissue regeneration treatment alternatives. Customizable GO-doped gelatine-based artificial tissue scaffolds with hierarchical structures were fabricated by SLA printing [222]. This scaffolds greatly promoted the glycosaminoglycan and collagen levels after GO-induced chondrogenic differentiation of human mesenchymal stem cells. Printed graphene composites consisting of majority graphene and minority polylactide-*co*-glycolide were also demonstrated to be able to support human mesenchymal stem cell adhesion, viability, proliferation, and neurogenic differentiation with significant upregulation of glial and neuronal genes [223]. Scaffolds decorated by magnetized graphene exhibited enhance biological functions and supported bone mesenchymal stem cell differentiation in vitro [224]. These studies confirm the significance and contribution of 3D printing and graphene in the area of bone tissue engineering.

The 3D printed graphene/polymer scaffold are also found to be promising in antibacterial applications. A polymer scaffold that was reinforced with silver nanoparticle decorated GO sheets realized sustained release of Ag ions from the scaffold, demonstrating an antibacterial performance (an antibacterial rate of 95% against Staphylococcus aureus) [225]. The GO sheets here provided sufficiently large specific areas for the in situ growth of Ag nanoparticles. Electroactive polycaprolactone scaffolds with conductive thermally reduced GO nanoparticle additives were fabricated by a 3D printing method, which showed a strong antibacterial effect which completely eradicated *S. aureus* on the surface of scaffold under direct current [226].

Graphene/polymer composite with well-defined structures fabricated by 3D printing have also been explored for many other bio-applications. For example, 3D printed graphene/PMMA composites with customized shape and good antimicrobial, stiffness and strength properties were proposed to be suitable for dental repair [227]. An autoclavable corona virus disease-19 face shield framework was fabricated by FDM printing of PLA composites with 1 wt% carbon fiber particle and 1 wt% graphene [228]. Incorporating graphene nanosheets into PLA filaments enabled the fabrication of 3D-printed devices capable of sterilization through exposure to near-infrared light at a power density equivalent to sunlight. This innovative approach can effectively eliminate viral particles, including those of the severe acute respiratory syndrome coronavirus, from the surface of 3D-printed objects within just 3 min of exposure [229]. Misra et al. developed a technology prototyped for personalized stenting based on 3D-printing of graphene/poly(ε-caprolactone) composites with dual drug incorporation to achieve controlled release of combinatorics as anticoagulation and anti-restenosis agents [230]. Conductive, 3D flexible graphene/poly(trimethylene carbonate) composite scaffolds with excellent compatibility with mesenchymal stem cell was proposed to be promising biomaterials to be applied as versatile platforms for biomedical applications [231].

## 4. Conclusions and Perspectives

In this review, we provide an overview of recent advances in 3D printed graphene and graphene/polymer composites. The commonly applied additive manufacturing techniques, including extrusion-based methods (DIW and FDM), photopolymerization strategies (SLS and DLP), and the powder-based technique (SLS), for graphene and its polymer-based composites are comprehensively reviewed. The unique combination of graphene’s exceptional properties and the design flexibility of 3D printing opens up exciting opportunities for the development of advanced materials with a wide range of functionalities. The ability to tailor the structural characteristics and properties of the 3D printed architectures enables the creation of custom-designed components for various fields, including energy storage, sensor, stretchable conductor, EMI shielding and bio-applications. Even though 3D printed graphene and graphene/polymer composites have been demonstrated to be promising in multifunctional applications, there still are important issues as follows to be addressed to unlock their full potential.

Owing to the layer-by-layer deposition fabrication process, the interlayer strength of 3D printed materials remains a critical aspect affecting their mechanical integrity and overall performance. Inadequate interlayer adhesion can lead to delamination, reduced strength, and compromised functionality, limiting the practical application of 3D printed graphene composites. More attentions on enhancing the interfacial strength of 3D printed objects are essential. Several strategies can be employed to enhance interlayer adhesion. First, optimizing print parameters, including temperature, layer height, and printing speed, is essential to achieve uniform and strong interlayer bonding. Ensuring consistent printing conditions reduces the risk of weak interfaces and delamination. Second, surface modification of graphene or polymer materials can improve their compatibility, promoting better interfacial bonding between adjacent layers. Third, the incorporation of interlayer bonding agents or adhesion promoters during the printing process can strengthen interlayer connections and enhance the overall adhesion. Additionally, post-processing techniques, such as annealing or surface treatments, are also promise in promoting interlayer bonding and improving the mechanical properties of 3D printed composites. A comprehensive understanding of the factors influencing interlayer strength and the implementation of these strategies will advance the field of 3D printed graphene composites.

As for 3D printed graphene-based materials, the diversity in materials, structures and functionalities is not fully explored yet. The 3D printing technique, with its ability to integrate multifunctional materials and complex structures seamlessly, can enable the creation of all-in-one customized devices with unprecedented capabilities. By incorporating different types functional materials, microscopic and macroscopic structures, the 3D printed composites can be endowed with integrated functionalities, meeting several or all specific requirements in a particular application. For example, with rational design of the material and structure, multifunctional sensors capable of detection and discrimination of different physical stimuli (such as temperature, pressure, tensile strain, bending, and moisture) are possibly be integrated by 3D printing techniques. The integration and de-coupling of multifunctional materials provides opportunities for graphene-based materials to be applied in complex situations.

In the realm of 3D printed graphene and graphene/polymer composites, the advantages stemming from the flexibility in structural and material design have only scratched the surface of their true potential. While researchers have made significant strides in leveraging this unique capability, there are vast unexplored opportunities awaiting discovery. The ability to tailor the structural characteristics and properties of these composites provides unmatched versatility, allowing the creation of custom-designed components for a wide range of applications. However, the full extent of this design freedom remains untapped. As 3D printing technologies evolve and our understanding of graphene’s behavior improves, we can expect groundbreaking advancements in the field. By exploring innovative material combinations, advanced additive manufacturing techniques, and optimized print parameters, we will witness the emergence of novel multifunctional materials with exceptional mechanical, electrical, thermal, and even biological properties.

## Figures and Tables

**Table 1:** Comparisons between different 3D printing techniques for graphene and graphene/polymer composites.

**3D Printing Techniques**	**Extrusion Based**		**Photopolymerization Based**		**Powder-Based**
	**DIW**	**FDM**	**SLA**	**DLP**	**SLS**
Applicable for graphene	Yes	No	No	No	No
Applicable for graphene/polymer composites	Yes	Yes	Yes	Yes	Yes
Printable materials	Viscous ink	Continuous filament	Photocurable slurry	Photocurable slurry	Powder
Graphene distribution	Uniform	Uniform	Uniform	Uniform	Segregated
Molding method	Solvent evaporation or freeze drying	Melting and cooling	UV laser-induced photocuring	UV light-induced photocuring	Laser-induced sintering
Characteristics of printed skeletons	Mainly in-plane framework	Mainly in-plane framework	Unrestricted	Unrestricted	Unrestricted
Efficiency	Relatively low	Decent	High	Very high	High

## Data Availability

Not applicable.

## Abbreviations

3D	Three-dimensional
DIW	Direct ink writing
FDM	Fused deposition modeling
SLA	Stereolithography
DLP	Digital light processing
SLS	Selective laser sintering, SLS
GO	Graphene oxide
PAA	Poly (amic acid)
ABS	Acrylonitrile butadiene styrene
PLA	Polylactic acid
rGO	Reduced graphene oxide
UV	ultraviolet
DMD	Digital micromirror device
LIG	Laser-induced graphene
PVDF	Polyvinylidene fluoride
PI	Polyimide
3DP-NGA	3D-printed N-doped graphene microlattice aerogel
CNT	Carbon nanotube
TPU	Thermoplastic polyurethane
PDMS	Polydimethylsiloxane
CNF	Carbon nanofiber
LED	Light-emitting diode
EMI	Electromagnetic interference shielding
SE	Shielding effectiveness
CIG	Carbonyl-iron and graphene
PMMA	Poly (methyl methacrylate)
PU	Polyurethane
